# Supplementary material for: Vancomycin heteroresistance (hVISA) in MRSA links to treatment failure and supports a revised PAP-AUC threshold
Source: Nat Commun. 2025 Dec 12;16:11251. doi: 10.1038/s41467-025-66118-8 (PMC12717272; doi:10.1038/s41467-025-66118-8)
Supplement: Supplementary file 1 — Supplementary Information [file 41467_2025_66118_MOESM1_ESM.pdf]

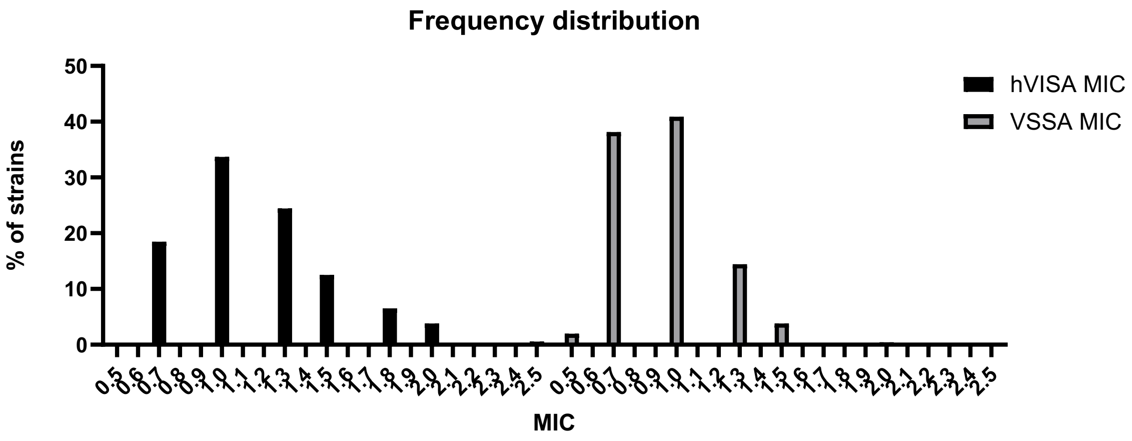


*Supplementary Figure 1: The distribution of VAN MIC values performed calculated by broth microdilution (mg/L) for hVISA strains (black) and VSSA strains (grey). The distributions are statistically different (t test p=0.001) indicating that the VAN MIC is an influencing factor to a strain being heteroresistant to Vancomycin.*
